# Test Bench for Right Ventricular Failure Reversibility: The Hybrid BiVAD Concept

**DOI:** 10.3390/jcm12247604

**Published:** 2023-12-10

**Authors:** Vincenzo Tarzia, Matteo Ponzoni, Demetrio Pittarello, Gino Gerosa

**Affiliations:** 1 Cardiac Surgery Unit, Department of Cardiac, Thoracic, Vascular Sciences and Public Health, University of Padua, 35121 Padua, Italy; ponzonima@gmail.com (M.P.); gino.gerosa@unipd.it (G.G.); 2 Institute of Anesthesia and Intensive Care, Padua University Hospital, 35128 Padua, Italy; demetrio.pittarello@aopd.veneto.it

**Keywords:** biventricular shock, BiVAD, Impella 5.5, ProtekDuo, right ventricular failure

## Abstract

Background: When heart transplantation and myocardial recovery are unlikely, patients presenting with biventricular cardiogenic shock initially treated with extracorporeal membrane oxygenation (ECMO) may benefit from a mechanical support upgrade. In this scenario, a micro-invasive approach is proposed: the combination of the double-lumen ProtekDuo cannula (Livanova, London, UK) and the Impella 5.5 (Abiomed, Danvers, MA) trans-aortic pump that translates into a hybrid BiVAD. Methods: All consecutive ECMO patients presenting with biventricular cardiogenic shock and ineligibility to heart transplantation from August 2022 were prospectively enrolled. The clinical course, procedural details, and in-hospital events were collected via electronic medical records. Results: A total of three patients, who were temporarily not eligible for heart transplantation or durable LVAD due to severe acute pneumonia and right ventricular (RV) dysfunction, were implanted with a hybrid BiVAD. This strategy provided high-flow biventricular support while pulmonary function ameliorated. Moreover, by differentially sustaining the systemic and pulmonary circulation, it allowed for a more adequate reassessment of RV function. All the patients were considered eligible for isolated durable LVAD and underwent less invasive LVAD implantation paired with a planned postoperative RVAD. In all cases, RV function gradually recovered and the RVAD was successfully removed. Conclusions: The Hybrid BiVAD represents an up-to-date micro-invasive mechanical treatment of acute biventricular failure beyond ECMO. Its rationale relies on more physiological circulation across the lungs, the complete biventricular unloading, and the possibility of including an oxygenator in the circuit. Finally, the independent and differential control of pulmonary and systemic flows allows for more accurate RV function evaluation for isolated durable LVAD eligibility reassessment.

## 1. Introduction

Biventricular cardiogenic shock is usually treated with extracorporeal membrane oxygenation (ECMO) in the very acute phase. However, the subsequent clinical decision should be taken promptly, since after 9 days of ECMO support, the patient’s prognosis changes dramatically [1]. Given the actual unavailability of total artificial hearts for most patients (due to the withdrawal of SynCardia and the significant body size limitations of the Aeson device), in case of ineligibility for heart transplantation or the unlikeliness of myocardial recovery, an upgrade from ECMO to a medium-term mechanical circulatory support (MCS) should be considered as a bridge-to-decision. Traditionally, extracorporeal ventricular assist devices (VAD) with a magnetically levitated centrifugal pump (Levitronix CentriMag, Levitronix LLC, Waltham, MA, USA) have been extensively used to sustain the patient’s hemodynamics until definitive treatment is achieved [2,3,4,5,6]. With respect to ECMO, biventricular VAD (BiVAD) generates a physiological circulation across the lungs and guarantees optimal left ventricular (LV) unloading [3], minimizing the risk of pulmonary stasis and edema related to inadequate LV unloading during ECMO support [7,8]. A correct LV unloading was demonstrated to improve coronary collateral flow in ischemic preclinical models, limiting myocardial injury and preserving ventricular function [9]. We have previously documented that a BiVAD configuration is particularly valuable in the setting of high-flow MCS, where the limitations of conventional ECMO are magnified [3]. Moreover, a BiVAD allows for a differential evaluation of LV and right ventricular (RV) function in order to assess the reversibility of RV failure once the LV is assisted, which would permit a durable LVAD implantation only.

The standard BiVAD implantation technique requires a full sternotomy, mainly to expose the pulmonary artery, which is hardly approachable through peripheral accesses. In addition, in the case of RV recovery, the removal of the right-sided VAD (RVAD) necessitates surgical re-entry. De Silva et al. proposed an innovative cannulation method for the RVAD outflow, which entails the use of a tunneled prosthetic graft that allows for a closed-chest decannulation [10]. The prolonged retention of foreign material connected to the circulatory system in the subcutaneous tissues is not a negligible drawback of this technique.

To completely obviate the need for a sternotomy, other less invasive strategies have emerged in recent years. Physiologic antegrade blood flow both in the systemic and pulmonary circulations, together with true RV and LV unloading, can be obtained by simultaneously implanting the Impella RP pump (Abiomed, Danvers, MA, USA) for RV support and the traditional Impella trans-aortic pump [11]. Known as “Bipella” [12], this promising approach has the potential to answer most of the needs of the ideal mechanical support device for the treatment of acute biventricular failure [13]. Unfortunately, this configuration cannot provide blood oxygenation, which can be severely impaired in the context of a cardiogenic pulmonary edema, and the Impella RP pump is approved for a maximum of 14 days of support, which limits its medium-term applications [14].

In this scenario, a further BiVAD strategy can be achieved by combining the double-lumen ProtekDuo cannula (Livanova, London, UK) and the Impella 5.5 (Abiomed, Danvers, MA) trans-aortic pump, enabling complete and differential biventricular support, RV and LV unloading, blood oxygenation, and high-hemocompatibility devices approved for medium-term support (30 days). This innovative hybrid percutaneous micro-invasive paradigm [15] has recently been raised as a state-of-the-art MCS configuration for acute biventricular failure.

We herein propose a novel protocol to manage patients with biventricular cardiogenic shock and severe respiratory impairment who are ineligible for heart transplantation and temporarily do not match the implantation criteria for a durable LVAD only. Our strategy entails an upgrade from ECMO to a medium-term hybrid BiVAD support as a bridge to heart transplantation/durable LVAD eligibility reassessment. The feasibility of the protocol and preliminary results from the first three patients treated with this approach are presented.

## 2. Materials and Methods

### 2.1. Protocol

**Figure 1:**
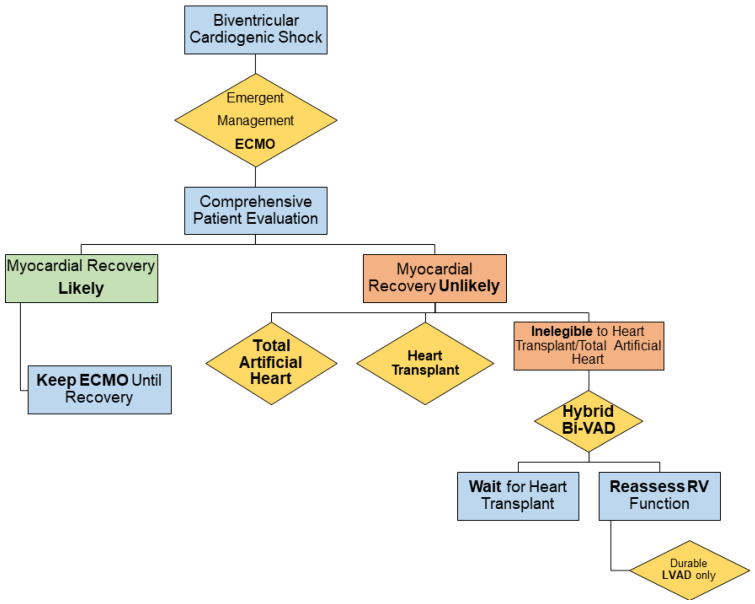
Decisional algorithm for current management of biventricular cardiogenic shock. BiVAD: biventricular assist device; ECMO: extracorporeal membrane oxygenation; LVAD: left ventricular assist device; RV: right ventricle.

As first-line therapy of biventricular cardiogenic shock, emergent circulatory support, is provided by peripheral venous-arterial ECMO. After the stabilization of the patient’s hemodynamics, a comprehensive evaluation is performed to assess the likelihood/unlikelihood of myocardial recovery based on clinical history, the etiology of heart failure, residual cardiac function at echocardiography, and the required mechanical/inotropic support to sustain the circulation. When myocardial recovery is considered unlikely, the next clinical decision should be ideally taken within 9 days from ECMO instauration, when we previously documented a significant shift in the patient’s prognosis [1]. In case of ineligibility (temporary or permanent) for heart transplantation and total artificial heart or low predicted chances of a matching donor (i.e., extreme ends of body surface area, “sensitized” recipient, rare blood group, et cetera) [16,17], the patient is considered for an upgrade to a hybrid BiVAD. After the implantation, if the underlying cause of ineligibility for heart transplantation is addressable, a specific treatment is pursued. In addition, a reassessment of RV function during differential RV and LV mechanical support is performed (full-flow LV support and low-flow RV support) to define the possibility of an isolated durable LVAD implantation. Figure 1 summarizes the clinical decision-making protocol.

### 2.2. Hybrid BiVAD Implantation Technique

The patient is brought to an operating room equipped with fluoroscopy and transesophageal echocardiography. The right axillary artery is exposed through a 5–6 cm incision and unfractionated heparin is administered to achieve an activated clotting time of >200 s. A 10 mm vascular prosthesis is anastomosed end-to-side to the axillary artery. The wire is introduced through the prosthesis and, under fluoroscopy guidance, the aortic valve is crossed. The Impella 5.5 is advanced and positioned with the inlet 5 cm below the aortic valve.

**Figure 2:**
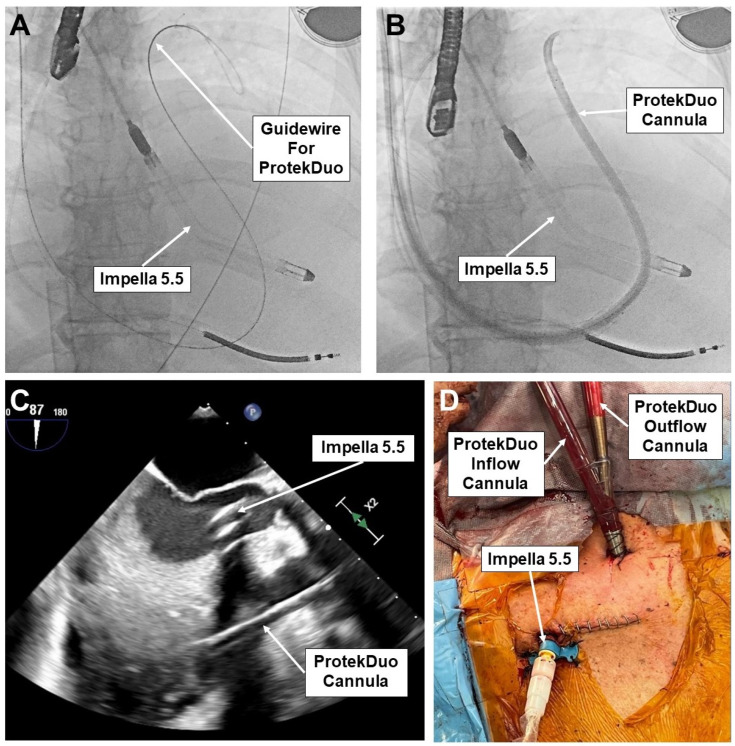
Fluoroscopy image of Impella 5.5 in place and advancing of a guidewire into the pulmonary artery for the ProtekDuo cannula (**A**). Fluoroscopy image of Impella 5.5 and ProtekDuo cannula (with introducer) in place (**B**). Transesophageal echocardiographic view of both Impella 5.5 and ProtekDuo cannula in place (**C**). Intraoperative view after completion of the procedure and instauration of Hybrid BiVAD support (**D**).

The ProtekDuo cannula is inserted percutaneously in the right jugular vein and advanced across the pulmonary valve, using fluoroscopy and transesophageal echocardiography [18] (Figure 2). The cannula is connected to the circuit of a third-generation magnetically levitated continuous flow pump which can be equipped with an oxygenator, depending on the grade of the respiratory compromise of the patient. Fully hemodynamic support is thus provided: up to 4.5 lpm for RV assistance with ProtekDuo and up to 6 lpm for systemic support with Impella 5.5.

### 2.3. Postoperative Management

After the hybrid BiVAD implantation, the inotropic support can be gradually decreased: dobutamine and epinephrine are maintained at low levels (dobutamine 3–5 mcg/kg/min; epinephrine 0.01–0.02 mcg/kg/min) and norepinephrine is tailored according to the grade of the vascular reactivity of the patient (0.01–0.05 mcg/kg/min). Diuretics are generously used to reduce pulmonary edema and optimize respiratory function until the oxygenator can be removed from the circuit and the patient can be extubated safely. Subsequently, RV support can be gently decreased by 0.5–1 lpm/day and transthoracic echocardiography is performed simultaneously to assess RV function. Moreover, the Impella 5.5 is equipped with an optical pressure sensor that, together with the novel Smart Assist technology, can detect real-time changes in LV end-diastolic pressure and cardiac output to further assist in defining RV performance.

Right ventricular qualitative function, shape, dimensions, and the degree of tricuspid valve insufficiency during RV support < 1 lpm are used to define the patient’s eligibility to isolated durable LVAD implantation and anticipate the risk of post-implantation RV failure, as we previously described [18]. Briefly, if the patient presents (during RV support <1 lpm) mild qualitative RV dysfunction, a triangular RV shape, a right-convex interventricular septum, and trivial/mild tricuspid regurgitation, a standard LVAD implantation is scheduled. Conversely, if qualitative RV function is moderately depressed, the RV is of triangular shape but dilated, the interventricular septum is flat, and tricuspid regurgitation is moderate or more, then the patient undergoes LVAD implantation as per our Planned Combo Strategy [18]. In case of severe RV dysfunction with severe RV dilatation (and loss of triangular shape) and torrential tricuspid valve regurgitation, the patient is not considered eligible for durable LVAD only.

**Figure 3:**
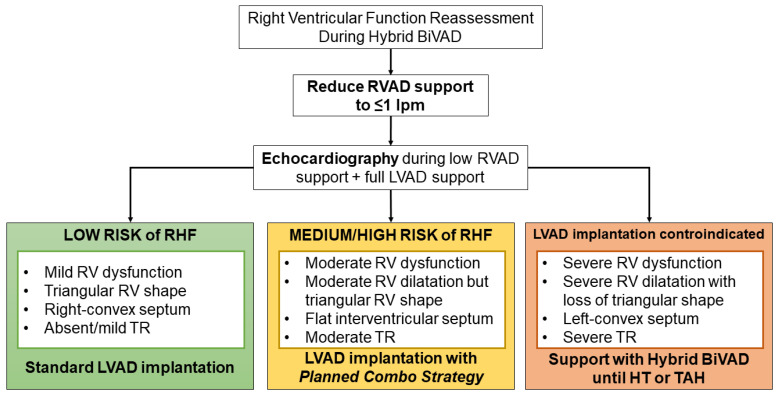
Decisional algorithm for RV function reassessment and durable LVAD eligibility evaluation during Hybrid BiVAD support. BiVAD: biventricular assist device; RVAD: right ventricular assist device; RHF: right heart failure; LVAD: left ventricular assist device; RV: right ventricle; TR: tricuspid regurgitation; HT: heart transplantation; TAH: total artificial heart.

According to our Planned Combo Strategy, the ProtekDuo cannula can be maintained during and after the durable LVAD implantation for a planned postoperative RVAD in those selected patients at higher risk of right heart failure [18]. Consequently, the risk of unexpected postoperative RV failure is contained, positively impacting the patient’s outcomes [19,20,21]. Durable LVAD implantation is achieved through less invasive accesses preferentially [22], which we documented can ameliorate the patient’s survival and reduce postoperative adverse events [23,24]. Subsequently, when RV complete recovery occurs, the ProtekDuo decannulation is accomplished bedside, with only one single deep hemostatic stitch. Figure 3 schematizes the protocol for RV function reassessment during hybrid BiVAD support.

### 2.4. Patients

All consecutive ECMO patients presenting with biventricular cardiogenic shock and ineligibility to heart transplantation from August 2022 were prospectively enrolled and evaluated according to the novel protocol. Demographic and clinical characteristics, procedural details, and in-hospital events were collected prospectively via electronic medical records. The local ethics committee approved the study (protocol 39680) and the patient’s informed consent was obtained.

## 3. Results

**Figure 4:**
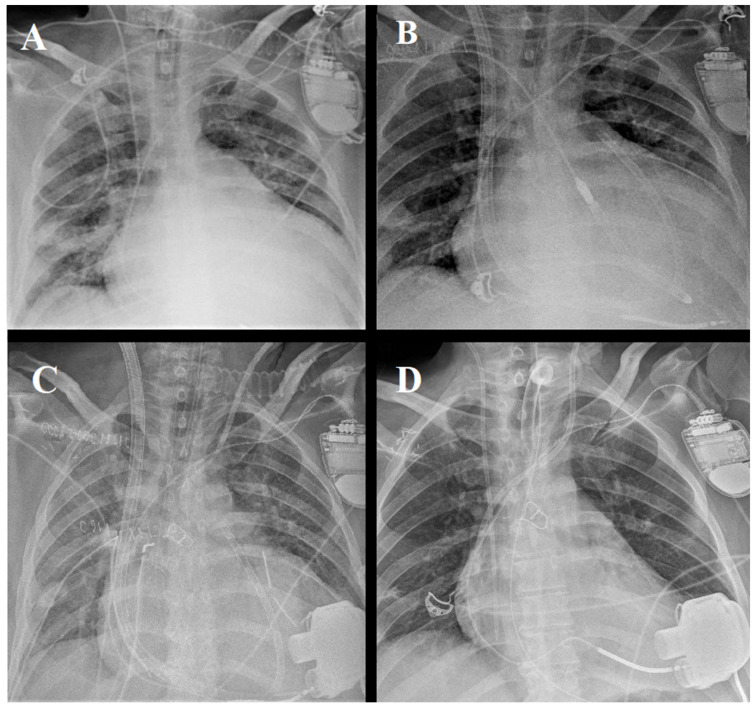
Patient #1 with biventricular cardiogenic shock and acute pneumonia on ECMO (**A**). Same patient on hybrid BiVAD (**B**), then after durable LVAD implantation with concomitant temporary RV support with ProtekDuo (**C**), and after ProtekDuo removal (**D**).

From August 2022, a novel protocol to manage ECMO patients with biventricular cardiogenic shock was introduced (Figure 1). A total of three patients presented with cardiogenic shock (due to ischemic dilated cardiomyopathy in two cases and idiopathic dilated cardiomyopathy in one) with severe biventricular dysfunction (LV ejection fraction <20% in all cases, and RV fractional area change of 20%, 12%, and 21%, respectively). The patients were initially managed with multiple inotropic agents and, due to progressive hemodynamic deterioration, they were subsequently treated with venous-arterial ECMO (STS-INTERMACS class 1). In addition, they presented with severe acute pneumonia of fungal etiology in two cases and viral in one (Figure 4), which contraindicated heart transplantation or durable LVAD implantation (Table 1).

According to our protocol, the three patients underwent ECMO upgrading to hybrid BiVAD after 4, 6, and 5 days of ECMO support, respectively. Combining the percutaneous ProtekDuo cannula (implemented with an oxygenator) and the Impella 5.5 in a hybrid BiVAD concept, we achieved a valid medium-term bridge-to-decision support while pulmonary function ameliorated. Intravenous therapy with antifungal or antiviral drugs (according to the isolated pathogens), as well as broad-spectrum antibiotics, was administered. The transition from ECMO to hybrid BiVAD support enabled more physiological hemodynamics and reliable ventricular unloading.

Given the presence of additional and not immediately addressable exclusion criteria to heart transplantation (fungal pneumonia and multi-drug-resistant A. baumanii respiratory tract colonization in patient #1, fungal pneumonia #2, and viral pneumonia and severe obesity in patient #3) and the irreversibility of LV failure, the patients were reassessed for isolated durable LVAD implantation.

During hybrid BiVAD, LV assistance was maintained at full flow, while RV support was progressively reduced (−0.5 lpm/day), with a concomitant titration of inotropes to maintain a stable Impella 5.5 flow and hemodynamics (target mean systemic arterial pressure > 60 mmHg). Transthoracic echocardiography was performed at every change of the RV support flow to monitor the RV response to an increased preload. Once RV support reached <1 lpm, RV function, shape, and dimensions were assessed by echocardiography to distinguish irreversible vs. reversible RV failure (Figure 3). By mimicking the hemodynamics status of durable LVAD implantation, we were able to anticipate the risk of RV dysfunction when the RV is paired with a normal LV cardiac output provided by the Impella 5.5.

After this reassessment, all patients were considered eligible for durable LVAD implantation, although with a high risk of post-implantation RV failure. In fact, during low-flow RV and full-flow LV support, two patients presented with mild RV dysfunction and mild RV dilatation, while one patient presented with moderate RV dysfunction and dilatation. In all cases, the RV preserved a triangular shape and the patients necessitated a low inotropic support to sustain the hemodynamics.

The patients underwent a less invasive LVAD implantation (bi-thoracotomy approach) with a Planned Combo Strategy [18]. After LVAD implantation, RVAD support with the ProtekDuo cannula was maintained to provide stable postoperative hemodynamics. As done previously during the hybrid BiVAD support, RV assistance was progressively reduced (−0.5 lpm every 1–2 days) under echocardiographic monitoring and the ProtekDuo cannula was successfully removed in all cases (after 28, 5, and 11 days from durable LVAD implantation, respectively), without recurrence of RV failure.

## 4. Discussion

Surgical options to treat biventricular cardiogenic shock beyond ECMO usually include heart transplantation, total artificial heart devices, and other forms of medium-term MCS. Nowadays, total artificial hearts are not available for the vast majority of patients due to the withdrawal of SynCardia (SynCardia Systems, LLC, Tucson, AZ, USA) and the significant body size limitations of the new Aeson device (Carmat, Velizy-Villacoublay, France). Moreover, heart recipients always exceed the number of possible donors, despite current investigations to expand the donor pool [25], and not every patient can be an eligible candidate for heart transplantation.

After the first 9 days of ECMO support, the patient’s survival displays significant attrition [1], especially if MCS is not able to preserve the multi-organ status adequately [26]. When myocardial recovery is unlikely and the patient possesses temporary or absolute contraindications to heart transplantation, an MCS upgrade should be planned. We previously documented that the required MCS flow to sustain the circulation impacts the patient’s prognosis directly [1,3]. In the cohort of patients necessitating a full-flow ECLS, we observed better clinical outcomes in those patients treated with BiVAD than conventional ECMO [3]. The two key aspects that differentiate the standard ECMO configuration and the BiVAD are the possibility of complete biventricular unloading and the instauration of physiologic systemic and pulmonary circulations [7,8,9,27]. These features of the BiVAD strategy become particularly significant in the context of biventricular cardiogenic shock with severely depressed myocardial contractility, necessitating high-flow circulatory support. In this scenario, LV distension is a serious threat that could further compromise the residual myocardial function and lead to apical thrombosis [28]. Moreover, when RV performance is also affected, the antegrade blood flow across the lungs is limited during the ECMO cannulation, resulting in reduced pulmonary perfusion [29].

As an attempt to contain the invasiveness of the traditional implantation of extracorporeal BiVAD, the BiPella (Impella trans-aortic pump + Impella RP pump) [11,12,30] and, subsequently, the Hybrid BiVAD (Impella + ProtekDuo) [31,32] were proposed as alternative strategies (Table 2). We incorporated the latter configuration in a novel protocol to optimize the clinical decision-making in ECMO patients with biventricular cardiogenic shock who are currently not eligible for heart transplantation or definitive treatment (Figure 1).

**Figure 5:**
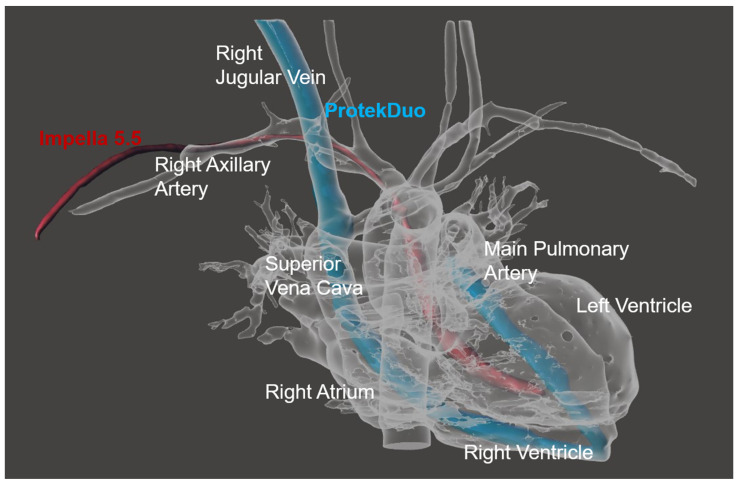
Three-dimensional reconstruction of a thoracic CT-scan of patient #3 while on hybrid-BiVAD support showing the Impella 5.5 pump (red) and the ProtekDuo cannula (blue).

By combining the ProtekDuo cannula and the most recent Impella 5.5 in a hybrid percutaneous micro-invasive concept (Figure 5), we achieved an up-to-date BiVAD that presents several key features: (1) complete biventricular support while maintaining extremely low invasiveness; (2) physiological flow patterns across the pulmonary and systemic circulations [3]; (3) full RV and LV unloading; (4) medium-term support (both devices are approved up to 30 days) and high grade of hemocompatibility; (5) possibility of including an oxygenator in the ProtekDuo circuit for respiratory support; and (6) differential RV and LV support to better assess the severity of RV dysfunction during LV assistance (Table 3).

Although the hybrid BiVAD requires a further surgical step (even though the ProtekDuo cannula is inserted percutaneously, which ensures extremely low invasiveness) than upgrading ECMO with the Impella pump only, it can provide antegrade pulmonary perfusion/oxygenation and more physiologic pulmonary circulation. These aspects become particularly valuable in the case of severely depressed myocardial function requiring high-flow MCS support and severe respiratory failure, with documented survival benefits [3]. Moreover, the hybrid BiVAD generates fully controllable distinct LV and RV flows, allowing differential ventricular loading/unloading and support. Finally, the hybrid BiVAD permits a straightforward adoption of the Planned Combo Strategy by maintaining the ProtekDuo cannula during and after durable LVAD implantation for a planned postoperative RVAD support. However, a direct and controlled comparison between the hybrid BiVAD and ECMO + Impella pump is still awaited.

Our preliminary data support the feasibility of this bridge-to-decision strategy, allowing the treatment of concomitant conditions (acute pneumonia) that were contraindicating a definitive surgical therapy for biventricular failure. We speculate that the upgrade from venous-arterial ECMO could have played a key role in achieving adequate LV unloading and avoiding pulmonary congestion related to left heart distension during ECMO [28]. This approach may have eased the recovery of pulmonary function from the infection. Moreover, since the patients displayed additional and not treatable exclusion criteria to heart transplantation, the hybrid BiVAD enabled a more accurate re-evaluation of RV performance, which was considered suitable for an isolated durable LVAD implantation in all cases. Since the risk of subsequent RV failure was considered high, we planned postoperative RVAD support by maintaining the ProtekDuo cannula during and after the durable LVAD implantation, according to our Planned Combo Strategy [18]. Although preliminary, our results suggest that the differential support of the systemic and pulmonary circulations allows for a more accurate reassessment of RV function during LV assistance. We consider the hybrid BiVAD (during full-flow Impella 5.5 support and low-flow ProtekDuo assistance) as the optimal test bench to evaluate the residual RV function to successfully couple with a durable LVAD implantation. Moreover, by adopting a less invasive approach and a planned postoperative RVAD, the risk of an RV failure relapse is minimized, allowing isolated LVAD implantation also in patients who were initially considered ineligible for further durable strategies.

## 5. Limitations

The present study represents a preliminary evaluation of the feasibility of our novel protocol for the clinical decision-making of patients with acute biventricular failure in whom heart transplantation or myocardial recovery cannot be achieved. We herein propose the hybrid BiVAD strategy as a novel treatment paradigm that exemplifies our efforts in pursuing the most up-to-date MCS technologies. The efficacy and safety of this strategy still need to be defined by a larger and controlled enrollment of patients. Particular caution should be paid in the interpretation and generalization of our findings until further data are available.

The evaluation of RV performance during Hybrid BiVAD support was performed using just qualitative echocardiographic metrics. The implementation of our decision-making algorithm with RV myocardial strain analysis (RV-free wall and global longitudinal strain) and the identification of quantitative cut-offs of RV function and dimensions represent the next directions of our investigations to further help patient stratification during Hybrid BiVAD support.

The comparison of the Hybrid BiVAD with other techniques of LV unloading during ECMO support requires further analysis.

## 6. Conclusions

The landscape of available technologies to treat cardiogenic shock is rapidly evolving. A cautious but creative combination of top-of-the-line devices may offer innovative solutions to extremely challenging clinical scenarios [55]. Although further investigations are still awaited to assess the safety of the hybrid BiVAD strategy, we believe that the concept of a “one ECMO fits all” now belongs to the past. A more physiologic, hemocompatible, and micro-invasive BiVAD support represents the frontiers of the treatment of biventricular cardiogenic shock, which can serve as the optimal test bench to evaluate the residual RV function for durable LVAD implantation eligibility reassessment.

## Figures and Tables

**Table 1:** Characteristics and outcomes of the first three patients treated with the hybrid BiVAD strategy.

Patient	Demographics	Heart Failure Etiology and Comorbidities	Pre-Implant Echocardiography	Exclusion Criteria to Heart Transplant	Mechanical Support Timeline	Hybrid BiVAD Maximal Flows	Complications during BiVAD	RV Reassessment during Low-Flow RVAD Support	Outcome
#1	44-year-old male	Idiopathic dilated cardiomyopathy; acute kidney injury	Severe biventricular dysfunction: LV ejection fraction 17%, RV fractional area change 20%, severe MR, mild TR	Acute pneumonia (Candida albicans), MDR respiratory tract colonization	ECMO (4 days) → Hybrid BiVAD (10 days) → Durable LVAD (Heartmate III) + ProtekDuo (28 days) → durable LVAD only	ProtekDuo: 3 lpm; Impella 5.5: 4.7 lpm	Nasopharyngeal bleeding	Moderate RV dysfunction, triangular RV shape, moderate RV dilatation, right-convex septum, moderate tricuspid regurgitation	Pulmonary recovery; durable intracorporeal LVAD + temporary (28 days) planned postoperative RVAD
#2	57-year-old male	Ischemic dilated cardiomyopathy; HCV hepatitis	Severe biventricular dysfunction: LV ejection fraction 18%, RV fractional area change 12%, severe MR, moderate TR	Acute pneumonia (Aspergillus fumigatus); persistence of respiratory tract fungal colonization	ECMO (6 days) → Hybrid BiVAD (15 days) → Durable LVAD (Heartmate III) + ProtekDuo (5 days) → durable LVAD only	ProtekDuo: 2.4 lpm; Impella 5.5: 4 lpm	Acute kidney injury requiring renal replacement therapy	Mild RV dysfunction, triangular RV shape, mild RV dilatation, right-convex septum, mild tricuspid regurgitation	Pulmonary recovery; durable intracorporeal LVAD + temporary (5 days) planned postoperative RVAD
#3	52-year-old male	Idiopathic dilated cardiomyopathy; diabetes mellitus	Severe biventricular dysfunction: LV ejection fraction 20%, RV fractional area change 21%, moderate MR, mild TR	Acute pneumonia (influenza virus type A and B), severe obesity (BMI: 36,9)	ECMO (5 days) → Hybrid BiVAD (19 days) → Durable LVAD (Heartmate III) + ProtekDuo (11 days) → durable LVAD only	ProtekDuo: 3.2 lpm; Impella 5.5: 4.5 lpm	Acute kidney injury requiring renal replacement therapy	Mild RV dysfunction, triangular RV shape, mild RV dilatation, right-convex septum, mild tricuspid regurgitation	Pulmonary recovery; durable intracorporeal LVAD + temporary (11 days) planned postoperative RVAD

BMI: body mass index; BiVAD: biventricular assist device; LV: left ventricle; LVAD: left ventricular assist device; MDR: multi-drug resistant; MR: mitral regurgitation; RV: right ventricle; RVAD: right ventricular assist device; TR: tricuspid regurgitation.

**Table 2:** Literature review: medium-term MCS strategies to treat acute biventricular failure beyond ECMO.

Device	Study (Year)	Number of Patients	Duration of Biventricular Support	Hemorrhagic Complications during Support	Thromboembolic Complications during Support	Outcome
Extracorporeal BiVAD (Centrimag eVAD)	Santise et al. (2006) [33]	2	2 and 7 days, respectively	Major bleeding in 1/2	None	Re-transplantation in 1/2 and myocardial recovery in 1/2
	John et al. (2007) [34]	12	Mean 9 days	-	-	Early mortality in 2/12, myocardial recovery in 2/12, and isolated durable LVAD in 8/12
	Shuhaiber et al. (2008) [35]	14	Mean 11 days	-	-	Early mortality in 9/14, heart transplantation in 3/14, myocardial recovery in 1/14, and isolated durable LVAD in 1/12
	Haj-Yahia et al. (2009) [36]	4	Mean 88 days	None	None	Heart transplantation in 4/4
	John et al. (2010) [37]	18	Mean 15 days	Hemolysis in 2/18	Stroke in 1/18	Early mortality in 10/18
	Mohite et al. (2013) [38]	21	-	-	-	Early mortality in 8/21, isolated durable LVAD in 6/21, and myocardial recovery in 7/21
	Mody et al. (2014) [39]	9	Mean 15 days	Intracranial bleeding in 1/9	-	Early mortality in 1/9, heart transplant in 1/9, and myocardial recovery in 7/9
	Takeda et al. (2017) [40]	90	Mean 24 days	Major bleeding in 59/90	Stroke in 13/90	Early mortality 19/90, myocardial recovery 25/90, heart transplantation 12/90, durable VAD 29/90
	Tarzia et al. (2022) [3]	34	Mean 11 days	Major bleeding in 16%	-	Early mortality 22%
Impella trans-aortic pump + Impella RP pump (Bipella)	Hunziker et al. (2013) [41]	1	-	-	-	Myocardial recovery
	Kapur et al. (2015) [42]	1	5 days	None	None	Isolated durable LVAD
	Aghili et al. (2016) [43]	1	3 days	None	None	Myocardial recovery
	Kamioka et al. (2017) [44]	1	4 days	Access hematoma	None	Extracorporeal BiVAD implantation
	Pappalardo et al. (2017) [12]	1	7 days	None	Limb ischemia	Myocardial recovery
	Kuchibhotla et al. (2017) [30]	20	Mean 5 days	Hemolysis in 6/20, major bleeding in 7/20	Limb ischemia in 1/20	Intra-hospital mortality in 10/20; myocardial recovery in 7/20 and isolated durable LVAD in 3/20
	Chiu et al. (2018) [45]	1	6 days	None	None	Myocardial recovery
	Dalal et al. (2019) [46]	1	3 days	Hemolysis	Limb ischemia	Myocardial recovery
	Ankola et al. (2020) [47]	3	Mean 13 days	Access bleeding in 2/3 and hemolysis in 2/3	None	Myocardial recovery in 3/3
	Karaaslan et al. (2021) [48]	1	3 days	None	None	Myocardial recovery
	Almejren et al. (2021) [49]	1	4 days	None	None	Myocardial recovery
	Puerto et al. (2021) [50]	1	5 days	Venous access bleeding and thrombocytopenia	None	Heart transplantation
	Caruso et al. (2021) [51]	1	3 days	None	None	Myocardial recovery
	Zoltowska et al. (2021) [52]	1	7 days	None	None	Myocardial recovery
	Ajello et al. (2022) [53]	1	10 days	Axillary access bleeding	None	Myocardial recovery
Impella + ProtekDuo cannula (Hybrid BiVAD)	Ruhparwar et al. (2020) [31] (Impella 5.0 and 5.5)	2	9 and 14 days, respectively	None	None	Isolated durable LVAD in 2/2
	Chivasso et al. (2021) [54](Impella CP)	1	14 days	None	None	Myocardial recovery
	Walsh et al. (2022) [32] (Impella 5.0)	13	-	None	RVAD thrombosis in 1/13	4/9 (31%) early mortality
	Current study (Impella 5.5)	3	Median 15 days	Minor bleeding in 1/3	None	Isolated durable LVAD in 3/3

**Table 3:** Key features of medium-term MCS devices for biventricular shock beyond ECMO. BiVAD: biventricular assist device; LV: left ventricle; RV: right ventricle.

Device	LV Unloading	RV Unloading	Physiological Flow	Low Surgical Invasiveness	Blood Oxygenation	Medium-Term Support (up to 30 Days)	Differential LV and RV Support	Possibility of Planned Combo Strategy
Traditional extracorporeal BiVAD (Centrimag eVAD)	✓	✓	✓	-	✓	✓	✓	-
Impella trans-aortic pump + Impella RP pump (Bipella)	✓	✓	✓	✓	-	-	✓	-
Venous-Arterial ECMO + Impella 5.5 pump	✓	✓	-	✓	✓	✓	-	-
Impella 5.5 pump + ProtekDuo cannula (Hybrid BiVAD)	✓	✓	✓	✓	✓	✓	✓	✓

## Data Availability

Data available upon request to the corresponding author.
